# Current and Future Nutritional Strategies to Modulate Inflammatory Dynamics in Metabolic Disorders

**DOI:** 10.3389/fnut.2019.00129

**Published:** 2019-08-26

**Authors:** Willem van den Brink, Jolanda van Bilsen, Kanita Salic, Femke P. M. Hoevenaars, Lars Verschuren, Robert Kleemann, Jildau Bouwman, Gabriele V. Ronnett, Ben van Ommen, Suzan Wopereis

**Affiliations:** ^1^Department of Microbiology and Systems Biology, Netherlands Organisation for Applied Scientific Research (TNO), Zeist, Netherlands; ^2^Department of Risk Analysis for Products in Development, Netherlands Organisation for Applied Scientific Research (TNO), Zeist, Netherlands; ^3^Department of Metabolic Health Research, Netherlands Organisation for Applied Scientific Research (TNO), Leiden, Netherlands; ^4^Janssen Research & Development, LLC, Spring House, PA, United States

**Keywords:** chronic low-grade inflammation, lifestyle, metabolism, phenotypic flexibility, resilience, nutrition

## Abstract

Obesity, type 2 diabetes, and other metabolic disorders have a large impact on global health, especially in Western countries. An important hallmark of metabolic disorders is chronic low-grade inflammation. A key player in chronic low-grade inflammation is dysmetabolism, which is defined as the inability to keep homeostasis resulting in loss of lipid control, oxidative stress, inflammation, and insulin resistance. Although often not yet detectable in the circulation, chronic low-grade inflammation can be present in one or multiple organs. The response to a metabolic challenge containing lipids may magnify dysfunctionalities at the tissue level, causing an overflow of inflammatory markers into the circulation and hence allow detection of early low-grade inflammation. Here, we summarize the evidence of successful application of metabolic challenge tests in type 2 diabetes, metabolic syndrome, obesity, and unhealthy aging. We also review how metabolic challenge tests have been successfully applied to evaluate nutritional intervention effects, including an “anti-inflammatory” mixture, dark chocolate, whole grain wheat and overfeeding. Additionally, we elaborate on future strategies to (re)gain inflammatory flexibility. Through epigenetic and metabolic regulation, the inflammatory response may be trained by regular mild and metabolic triggers, which can be understood from the perspective of trained immunity, hormesis and pro-resolution. New strategies to optimize dynamics of inflammation may become available.

## Introduction

Obesity, type 2 diabetes, and other metabolic disorders have a large impact on the health of society in Western and developing countries. These disorders not only increase the risk of cardiovascular disease; they also represent a common preventable cause of cancer (1, 2). Currently, about 15% of the world population suffers from obesity, 5–10% has type 2 diabetes, and these numbers are increasing (3, 4). An important hallmark of metabolic disorders is chronic low-grade inflammation, marked by histological changes of tissues and a phenotypic shift in immune cell types (5–7). Eventually, such tissues release pro-inflammatory cytokines, acute phase proteins, pro-inflammatory lipids, and other biological inflammatory mediators into the circulation, converting tissue-based low-grade inflammation into a systemic inflammatory condition (5, 8, 9).

To be able to quantify low-grade inflammation without biopsies before it progresses to a systemic disease, one of the current challenges is to identify systemic biomarkers that reflect local tissue inflammation caused by metabolic dysfunction (8, 9). As a solution, dynamic time-resolved metabolic responses to metabolic challenge tests (e.g., the oral glucose challenge test (OGTT) or mixed meal test), have been used to evaluate inflammation in conditions of metabolic dysfunction (10).

While current anti-inflammation therapies often focus on inflammation only, new possibilities may exist for the creation of interventions in the interaction between metabolism and inflammation dynamics. Increased anti-inflammatory nutritional intake [as defined by an anti-inflammatory diet potential, see (2)], for example, is associated with a decreased risk in overall mortality, cardiovascular disease, and cancer (2). Inflammation is seen as a pathological state resulting from many disease processes that occur late in pathogenesis and normally is targeted at a late stage in disease pathogenesis. To intercept this process before irreversible changes occur, a refocus is needed onto the early stages of low-grade inflammation. Since metabolism and the innate immune system are closely connected [for review, see (5, 7)], interception could focus on metabolism as well as on inflammation dynamics to restore disbalances.

In this review, we give a short introduction to the role of chronic low-grade inflammation in metabolic disorders. Next, we describe the state-of-the-art of metabolic challenge tests for the diagnosis of chronic low-grade inflammation, and the evaluation of nutritional interventions to reverse it. Additionally, we give a future perspective on innovative strategies that focus on the training of inflammatory dynamics to prevent or reverse chronic low-grade inflammation in metabolic disorders.

## Low-Grade Inflammation and Metabolic Control

Inflammation is a central part of the immune system as an attempt to heal the body after injury, defend itself against foreign invaders, and to repair damaged tissue. Healthy inflammation prevents wounds from persisting and infections from becoming lethal.

**Figure 1:**
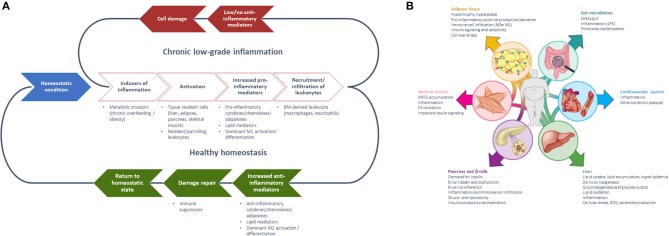
**(A)** Generalized model of sequential steps involved in the inflammatory response in metabolic tissues resulting in chronic low-grade inflammation or return to a healthy homeostatic condition [inspired by Villeneuve et al. (17)] and **(B)** Manifestation of chronic low-grade inflammation in the different metabolic tissues [Reprinted with permission from Ralston et al. (18)].

**Figure 2:**
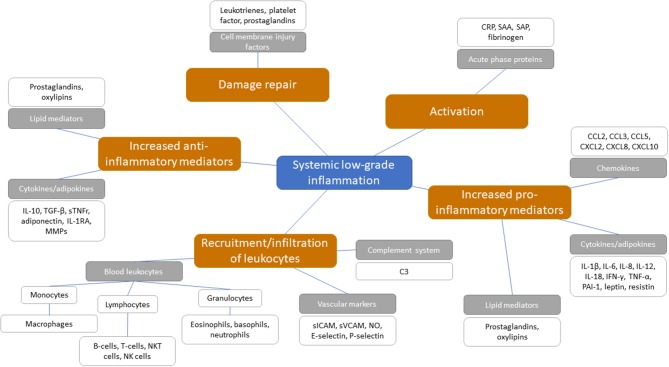
Potential systemic biomarkers categorized following the sequential steps involved in the inflammatory cascade with chronic low-grade inflammation. Orange boxes represent the sequential steps involved in the inflammatory response within chronic low-grade inflammation. Gray boxes represent inflammatory biomarker classes and are categorized under the sequential inflammatory steps. White boxes present the biomarkers as positioned under the inflammatory biomarker classes.

Inflammation may manifest in an acute, high-grade as well as in a chronic low-grade manner (Table 1). Acute inflammation is short-term and the effects subside after a few days once the damage/infection/irritation has been repaired or removed and mainly involves cells of the innate immune system. In contrast, chronic low-grade inflammation is long-term, and can constitute a damaging process with a mild sustained induction of inflammation. As a response to such an induction (e.g., metabolic stressor), tissue-resident cells and resident/patrolling leukocytes become activated, resulting in the secretion of pro-inflammatory mediators which leads to the recruitment/infiltration of pro-inflammatory leukocytes (11). Under healthy conditions, local immune cells such as macrophages and regulatory T cells produce anti-inflammatory cytokines that resolve inflammation and re-establish homeostasis (12, 13). However, without (or with insufficient) activation of anti-inflammatory or inflammation-resolving mediators, this results in the amplification of inflammation by autocrine and paracrine signals resulting in an imbalance of the immune regulatory network (14–16) (Figures 1A, 2).

**Table 1:** Features of acute and chronic low-grade inflammation [adapted from Calder et al. (9)].

	**Acute inflammation**	**Chronic low-grade inflammation**
Onset	Immediate	Delayed
Duration	A few days	Unlimited
Location	Typically local	Systemic, insulin dependent tissue
Trigger	Pathogens, injured tissue	Metabolic disturbance; some chronic infections
Inducer	PAMP and DAMP	Metabolic DAMP
Major cells involved	Neutrophils and other granulocytes, monocytes, macrophages; T cells later	Monocytes, macrophages, T cells, B cells, adipocytes
Primary mediators	Vasoactive amines, eicosanoids; chemokines and cytokines later	Cytokines, chemokines, adipokines, eicosanoids, reactive oxygen species, hydrolytic enzymes
Outcomes	Resolution, abscess formation, chronic inflammation	No overt pathology, tissue (vascular) damage, increased insulin resistance, intracellular lipid accumulation
Associated pathologies	Colitis, peritonitis, systemic inflammatory response syndrome (SIRS)	Dyslipidemia, atherogenesis, diabetes mellitus type 2, systemic arterial hypertension

*DAMP, damage-associated molecular patterns; PAMP, pathogen-associated molecular patterns*.

**Figure 3:**
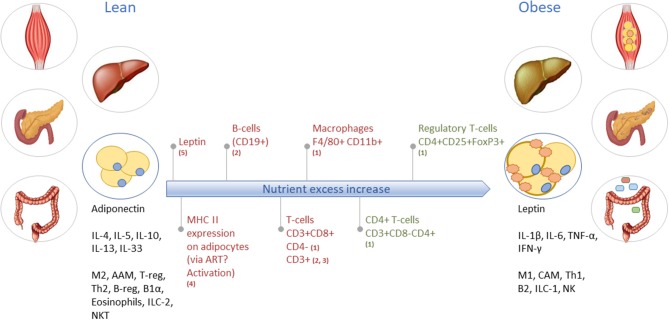
Obesity-induced development of tissue inflammation in various organs. As an example, the long-term inflammatory dynamics and characteristics of lean toward obese WAT are illustrated. In lean WAT, immune cells are generally in an overall Type2 state (left black text), while in obese WAT the immune cells operate in a Type1 state (right black text). Red text indicates increase/abundancy of the mentioned adipokine and immune cells. Green text represents a decrease in the mentioned immune cell. M2, macrophage type 2; AAM, alternatively activated macrophages; T-reg, regulatory T-cells; Th2, T-helper type 2 cells; B-reg, regulatory B-cells; B1α, B-cells type 1 alpha; ILC-2, innate lymphoid cells type 2; NKT, natural killer T-cells; M1, macrophage type 1; CAM, classically activated macrophages; Th1, T-helper type 1 cells; B2, B-cells type 2; ILC-1, innate lymphoid cells type 1; NK, natural killer cells. Based on (1) (21); (2) (22); (3) (23); (4) (24); and (5) (25).

The mechanistic basis for the interaction between metabolic and inflammatory systems is referred to as metaflammation [for review, see (5, 7)]. From an evolutionary perspective, the interaction between immune and metabolic pathways is highly conserved from invertebrates to mammals (5). The fat body of the fruit fly functions to sense and store nutrients, as well as to defend against pathogenic invaders (19). Throughout evolution, the fat body has developed into distinguishable metabolic and immune organs that exist in mammals, amongst which are adipose tissue and liver. Signaling pathways [e.g., NLRP3 inflammasome (18, 20), JNK-NFkB pathway (5)], however, are conserved within these tissues, so with either inflammation or metabolic dysfunction, the same pathways become activated (5). These mechanisms are ubiquitously present in all metabolic tissues and take part in the progression from a healthy to a metabolically compromised state (Figures 1B, 3). Nevertheless, tissue-specific characteristics of chronic low-grade inflammation also exist, which are described in more detail below (Figure 1B) (18).

*Adipose tissue* inflammation is characterized by hypoxia and mechanical stress following hypertrophy—and possibly also hyperplasia of fat tissue (26–29)—that causes adipocytes to change into a pro-inflammatory secretory profile of cytokines and adipokines (e.g., higher TNF-α and leptin, lower adiponectin, and resistin) (27). Furthermore, the metabolic balance within adipocytes shifts from glycolysis toward lipolysis, which leads to increased free fatty acid levels in the system that stimulate the cellular metaflammatory pathways (11, 28). *Intestinal* inflammation is associated with dysbiosis, i.e., the impaired ability of the microbiome-intestinal system to adapt to alterations (30). The breakdown of the human-microbiome partnership is able to negatively change the epithelial barrier function, as well as the production of specific metabolites and immune function (31, 32). Due to reduced barrier and adaptive and innate immune function, the translocation of bacterial endotoxins and dietary products from the gut into the systemic circulation is possible and can expand low-grade inflammation into other organs (33). The *liver* is exposed to these gut-derived products, which can lead to liver inflammation in metabolic syndrome (MetS) (34). Moreover, metabolic stressors (e.g., saturated fatty acids, glucose) activate hepatocytes to release alarmins (IL-33, HMGB1), extracellular vesicles that contain inflammatory effector proteins and microRNAs, hepatokines (fetuin-A, selenoprotein P), and other stress molecules such as ATP, ceramides and nitric oxide (18, 34–36). These stressors all activate a local inflammatory response via e.g., Kupffer cells (34). Investigations toward the initiation of *skeletal muscle* inflammation are ongoing, however, it appears that inflammation is mainly manifested in intermyocellular and perimuscular adipose tissue. Free fatty acids and inflammatory molecules from perimuscular adipose tissue and other tissues stimulate myocyte inflammation and dysregulated myocyte metabolism (20, 37, 38). The release of cytokines and myokines, in turn, contributes to insulin resistance and low-grade inflammation (37).

Given that such local tissue inflammation precedes the systemic manifestation of disease, it is a challenge to identify systemic biomarkers that represent local tissue inflammation related to metabolic dysfunction.

## Dysfunctional Lipid Control Mechanisms as the Basis of Low-Grade Inflammation

Lipid control is a key player in local tissue inflammation. There is a tight connection between metabolic and inflammatory pathways; they overlap and use the same master regulators (5, 7). This may explain that metabolic overload requires adjustment of regulators of lipid metabolism that inherently also affects inflammatory processes (39, 40).

One or more of the following four impairments in lipid control mechanisms may cause metabolic inflammation and activation of inflammatory pathways:

- Inappropriate adjustment of lipid uptake and lipid handling processes via the enterohepatic cycle involving the gut, microbiota and the liver (41–43);
- Inappropriate storage of excess lipids as ectopic fat in organs not designed for lipid storage (44, 45). In contrast to adipose tissue depots that can respond with hyperplasia, these depots have limited storage capacity and respond mainly in terms of hypertrophy. For example, mesenteric adipose tissue that has close anatomical proximity to the liver (45, 46);
- Intracellular imbalance between pro-inflammatory vs. anti-inflammatory lipids;
- Unfavorable composition of circulating lipids and high content of fatty acids that are prone to oxidation (47). The intracellular presentation of lipids determines accessibility and utilization within a cell, e.g., as small or large lipid droplet, and their role in the circulation, e.g., as part of VLDL/LDL lipoprotein or HDL lipoprotein (47, 48). For example, it is assumed that larger droplets are more detrimental and associated with tissue inflammation. Indeed, large macro-vesicular lipid droplets are often found in pathological conditions (e.g., in non-alcoholic fatty liver disease) and are associated with inflammation and fibrosis (47, 49).

There is a series of blood-based biomarkers that may reflect the different steps in chronic low-grade inflammation (Figure 2). These include well-known biomarkers, e.g., acute phase proteins, cytokine and chemokine levels, as well as some more innovative biomarkers, e.g., oxylipins, an oxidation product of essential fatty acids and related to vascular function and inflammation associated with metabolic function (50, 51). While some of these biomarkers (e.g., acute phase proteins and cytokines) are appropriate for measuring acute inflammation, no good systemic blood-based biomarkers exist for the identification of chronic low-grade inflammation on an individual level (8, 9, 52, 53). Although chronic low-grade inflammation in multiple organs can be present, this may not yet be detectable in the circulation (54).

## Measuring Phenotypic Flexibility for Early Detection of Metabolic Disorders

Phenotypic flexibility is referred to as the ability to maintain homeostasis by a highly energy-dependent, rapid and orchestrated adaptation to continuous changes of the environment, such as nutrient intake, physical exercise, and temperature changes. Phenotypic flexibility is the opposite of dysmetabolism, i.e., the inability to keep homeostasis, that eventually leads to e.g., oxidative stress, insulin resistance and dysbiosis (42, 55–59). Continued metabolic overload may eventually lead to chronic or permanent adaptation of the system, characterized by mitochondrial dysfunction and low-grade inflammation.

**Figure 4:**
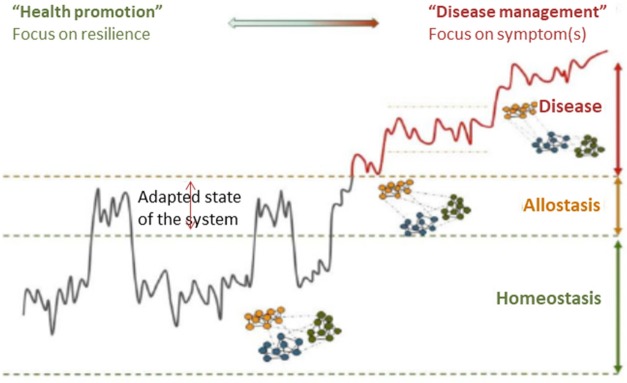
A framework for understanding the progression of healthy homeostasis to disease. For chronic diseases, the pathological development toward a disease state typically takes years. While the medical world has made tremendous progress toward the measurement of disease, the ability to measure the progress toward the disease is lacking. Resilience as measure of health status provides a promising solution to this problem. In contrast to the classical symptom/biomarker approach, challenge tests are used to measure resilience as an early biomarker of disease or (nutritional) effect. Reprinted with permission from van der Greef et al. (64).

**Figure 5:**
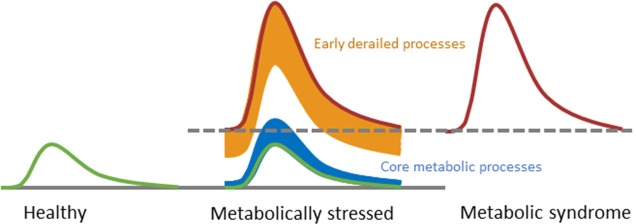
Conceptual representation of challenge tests may evaluate the progression toward metabolic disorder. During progression from a healthy state toward a state of metabolic syndrome, only the early derailed processes respond differently to a challenge test in order to keep the core processes in balance. Eventually, the balance of the core processes is also disrupted. Modified with permission from Kardinaal et al. (51).

The measurement of one's health status via phenotypic flexibility is possible by analyzing the dynamical responses to a metabolic challenge test (60–62). Considering that lipid control plays a key role in chronic low-grade inflammation, the response to a metabolic challenge containing lipids may magnify dysfunctionalities on the tissue level causing an overflow of inflammatory markers into the circulation and hence allow early detection of metabolic and inflammatory dysfunction in an individual via dynamical response profiles (61–63) (Figure 4). Early signs of reduced flexibility (or dysmetabolism) manifest through processes that are regulatory and adaptive in order to keep core metabolic processes in balance (51) (Figure 5). An example of an adaptive process is the production of insulin, which is increased under insulin resistance conditions in order to maintain the core process of glucose dynamics. The regulatory, adaptive and core metabolic processes are closely connected, shown by the myriad of dynamical biomarker responses to a metabolic challenge, including incretins, satiety markers, bile acids, amino acids, fatty acids and inflammatory markers [see (10, 61) for review]. Inflammatory markers do respond to glucose (65) and lipid challenge tests (66), as well as a combination of both (67, 68), albeit that only subtle effects were observed for inflammatory mediators in the circulation. This is not surprising given that inflammatory control is one of the core metabolic processes (51). Interestingly, several oxylipins, acute phase proteins, and vascular markers showed stronger responses to metabolic challenge tests (51, 68), marking them as potential early dynamical markers for metabolic stress.

With the aim to measure phenotypic flexibility by one standardized oral challenge, a systematic literature evaluation was previously performed to identify the optimal composition of a metabolic challenge test (10). A challenge test that combined glucose, proteins, and lipids in one drink was superior to those with fewer macronutrients. The systemic responses involved multiple local and systemic processes, including those from the gut, adipose tissue, muscle, liver, kidney, vasculature, pancreas, brain (10, 69). The work resulted in the standardized so-called “PhenFlex” oral challenge test, composed of 75 g glucose, 60 g palm olein (36.6% mono-unsaturated fatty acids, 48.8% saturated fatty acids, 9.1% poly-unsaturated fatty acids) and 20 g protein, enabling the comparison of phenotypic flexibility among studies and different stages of health (10, 54, 62).

Several studies have evaluated the potential of metabolic challenge tests for early diagnosis of chronic low-grade inflammation in the context of metabolic diseases (Table 2).

- In type 2 diabetes, both fasting and postprandial concentrations of inflammatory and vascular markers are higher as compared to healthy individuals (65, 74, 75). Furthermore, the post-prandial increase of pro-inflammatory mediators and decrease of anti-inflammatory mediators was stronger in type 2 diabetics (74), and it took longer for cytokines and vascular markers to return to baseline (75).
- In MetS vs. healthy subjects acute phase proteins were increased at fasting, while a challenge test was needed to reveal increased responses of cytokines and vascular markers and reduced responses of oxylipins (51).
- In obese subjects, fasting levels and challenge responses of acute phase proteins were increased as compared to healthy individuals (66, 72). In contrast to MetS, however, of the cytokines only TNF-α showed an increased response to a lipid challenge test (73). Other cytokines, vascular markers, and oxylipins showed no difference between obese and healthy persons (50, 66, 72, 73). Additionally, gene expression of platelet activation and human leukocyte antigen class II was increased, as well as the challenge test response of lymphocytes and neutrophils (71, 72).
- Having a high percentage of bodyfat in combination with aging leads to higher levels of pro-inflammatory cytokines, and lower levels of anti-inflammatory cytokines (70, 76).

**Table 2:** Metabolic challenge tests to diagnose chronic low-grade inflammation in metabolic disorders.

**Observation**	**Study design**	**Biomarkers**	**Reference**
Basal and inflammatory resilience was different between the two populations that served as “health reference groups”	Young and lean (20–29 years, low body fat, *n* = 20), and old and overweight (50–70 years, high body fat', *n* = 20) male and female subjects PhenFlex challenge test dynamics up to 4 h	TNF-α^^*^, ^#^^, IL-6^*^^, ^#^^, IL-8^*^^, ^#^^, **IL-10**^**^#^**^	(70)
No significant differences were observed in basal inflammatory state between lean and MetS subjects. Inflammatory resilience was reflected in diminished cytokine and oxylipin responses.	35–65 years old lean (*n* = 10) and MetS (*n* = 10) male subjects High fat challenge test dynamics up to 8 h	CRP, SAA, IL-1β, **IL-6**^**^#^**^, IL-8, IL-10, IL-12p70, **IL-18**^**^#^**^, TNF-α, IFN-γ, C3, sICAM1, sVCAM1, adiponectin, leptin^*^, resistin, **complement C3**^**^#^**^9(S)-HODE, 13(S)-HODE, 11(S)-HETE, 12(S)-HETE, 9,10-DiHOME, 12,13-DiHOME, **19,20-DiHoPE**^**^#^**^, **8,9-DiHETrE**^**^#^**^, **11,12-DiHETrE**^**^#^**^, 14,15-DiHETrELeukocytes, lymphocytes, neutrophils, monocytes, eosinophils, basophils	(51)
Lower basal antigen presentation to T-lymphocytes in obese vs. lean, decreased inflammatory resilience reflected increased PBMC gene expression responses to challenge associated with platelet activation.	50–70 years old lean (*n* = 17) and obese (*n* = 15) male subjects High fat challenge test dynamics up to 4 h	Genes belonging to: **human leukocyte antigen (HLA) class II (e.g.**, ***HLA-DQA2, HLA-DQB1, HLA-DRB5***)^*^, **platelet activation (e.g.**, ***PF4, PPBP, ITGA2B, ITGB3, SELP, PDE3A, CD9, GP1BA, GP6, GP9*****)**^**^#^**^	(71)
No differences were observed in basal levels or challenge induced levels of oxylipins between lean and obese subjects	50–70 years old lean (*n* = 18) and obese (*n* = 18) male subjects High fat challenge test dynamics up to 4 h	12(13)-EpOME, 12,13-DiHOME, 9(10)-EpOME, 9,10-DiHOME, 9,12,13-TriHOME, 13-HODE, 13-HpODE, 13-KODE, 9-HODE, 9-HpODE, 9-KODE, PGF1a, PGD2, PGE2, PGF2a, TXB2, 11-HETE, 12-HHTrE, 14,15-DiHETrE, 11,12-DiHETrE, 8,9-DiHETrE, 5,6-DiHETrE, 12-HETE, 15-HETE, 15-KETE, 5-HETE, 9-HOTrE, TXB3, 17,18-DiHETE, 14,15-DiHETE, 12-HEPE, 15-HEPE, 5-HEPE, 19(20)-EpDPE, 19,20-DiHDPA, 17-HDoHE	(50)
Basal CRP was different between lean and obese, but no postprandial effect observed	25–55 years old lean (*n* = 19) and obese (*n* = 17) male subjects High fat challenge test dynamics up to 6 h	CRP^*^, IL-6, endotoxin	(66)
Elevated basal inflammatory state in obese vs. lean reflected in higher acute phase protein and lower monocyte levels, decreased inflammatory resilience reflected in lower response of immune cells and cellular adhesion of lymphocytes.	50–70 years old lean (*n* = 18) and obese (*n* = 18) male subjects High fat challenge test dynamics up to 4 h	IL-6, IL-8, CRP^*^, SAA^*^, E-selectin, P-selectin, sICAM1, sICAM3, sVCAM1, Thrombomodulin, vWF^*^Neutrophils^**^#^**^, monocytes^*^^, ^#^^, **lymphocytes**^**^#^**^CD11a_N_, CD11b_N_, CD62L_N_, CD11a_M_, CD11b_M_, CD62L_M_, ${\text{CD11a}}_{L}^{\#}$, ${\text{CD11b}}_{L}^{\#}$, CD62L_L_	(72)
Elevated basal inflammatory state in obese/diabetic vs. lean reflected in cytokine levels, lower inflammatory resilience reflected in lower cytokine and higher PBMC gene expression responses to challenge	50–70 years old lean (*n* = 18), obese (*n* = 18) and obese/diabetic (*n* = 6) male subjects High fat challenge test dynamics up to 4 h	IL-1β^*^^, *a*^, **TNF-α^^#^^*****IL1b**^***^#^, a***^, IL8, **MCP1**^***^#^, a***^, NFκB1, TNFα*^a^Only significant for obese+diabetic vs. lean and obese	(73)
Both basal and challenge induced levels of cytokines and vascular markers discriminated between healthy volunteers and diabetic patients	Healthy (*n* = 256) and diabetic (*n* = 274) male and female subjects above 18 years (mean age 46 and 48, respectively) OGTT dynamics up to 3 h	CRP^*^^, ^#^^, IL-6^*^^, ^#^^, TNF-α^^*^, ^#^^, sICAM1^*^^, ^#^^, sVCAM1^*^^, ^#^^, sE-selectin^*^^, ^#^^	(65)
The basal inflammatory state is different between healthy and diabetic individuals, while the inflammatory resilience is different for IL-8.	Healthy (*n* = 30, mean age 40.5 years) and diabetic (*n* = 30, mean age 42.3) male and female subjects High carbohydrate, high fiber challenge test dynamics up to 4 h	IL-8^*^^, ^#^^, IL-18^*^, adiponectin^*^	(74)
Basal inflammatory state, as well as inflammatory resilience was different between healthy and diabetic subjects.	Healthy (*n* = 20, mean age 44 years) and diabetic (*n* = 20, mean age 46) male and female subjects High-fat challenge test dynamics up to 4 h	IL-6^*^^, ^#^^, TNF-α^*^^, ^#^^, sICAM1^*^^, ^#^^, sVCAM1^*^^, ^#^^	(75)

Markers that were statistically different between healthy/lean and metabolic disorder groups are shown in bold.

* marks a difference in fasting levels and

# *indicates a different response to the challenge test*.

Altogether, the application of a challenge test provides richer results contributing to a more comprehensive diagnosis of chronic low-grade inflammation in type 2 diabetes, MetS, and obesity.

## Measuring Phenotypic Flexibility to Demonstrate the Beneficial Effects of Interventions on Low-Grade Chronic Inflammation

Ultimately, it is desirable to develop nutritional intervention strategies to impact an individual's health status toward a healthier phenotype (59). Epidemiological studies have shown associations of improved nutritional habits with reduced inflammation, including a high unsaturated/saturated dietary fat balance, low dietary carbohydrates, dietary fibers, probiotics, vitamins, as well as flavonoids (8, 28, 52, 77–80). Other interventions, such as dietary supplements and weight loss can also significantly reduce levels of inflammatory markers (79, 81–83). Several studies have identified novel biomarkers that represent nutrient-induced modulation of inflammation indicating an anti-inflammatory effect (9, 53, 79, 84, 85). However, the subtle and presumably chronic effects of nutrition limit the substantiation of positive health effects in randomized clinical trials, regardless of the epidemiological and mechanistic evidence (86). Here, we summarize the current findings from applying metabolic challenge tests for evaluation of nutritional effects focusing on dynamics of inflammation (Table 3).

- An anti-inflammatory dietary mixture (AIDM) containing resveratrol, green tea extract, α-tocopherol, Vitamin C, n3-poly-unsaturated fatty acids and tomato extract showed improvement of inflammatory resilience in a 5-week randomized, double-blind crossover trial. Although fasting levels of sVCAM-1 and IL-18 decreased with the intervention, other cytokines, vascular markers, blood-clotting factors, and immune markers were found reduced post-prandially only (67, 79).
- Dark chocolate consumption was found having anti-inflammatory effects in a 4-week randomized clinical trial, which was especially visible in the reduced post-challenge responses of cytokines, vascular markers, white blood-cells and leukocyte-activation markers (87).
- Overfeeding of healthy males in a 4-week double-blind crossover study led to disturbed post-challenge dynamics of cytokine markers, white blood cells, vascular markers, and oxylipin markers (51). Interestingly, the inflammatory resilience phenotype of the overfed healthy males moved toward that of males diagnosed with MetS (51).
- Wholegrain vs. refined wheat intervention in a 13-week randomized, double-blind trial showed a beneficial effect on the post-prandial responses of acute phase proteins and cytokines, but not on their fasting levels (70).

**Table 3:** Metabolic challenge test to evaluate nutritional effects on chronic low-grade inflammation.

**Observation**	**Study design**	**Biomarkers**	**References**
Wholegrain wheat consumption leads to lower basal CRP and sICAM3 as compared to refined wheat consumption, whereas increased inflammatory resilience was observed for multiple cytokines.	Refined wheat (*n* = 25) vs. wholegrain wheat (*n* = 25) intervention in 45–70 years old males and females with elevated cholesterol (>5 mmol/L) PhenFlex challenge test dynamics up to 4 h	**TNF-α**^^#^^, **IL-6**^^#^^, **IL-8**^^#^^, **IL-10**^^#^^, CRP^*^^, ^#^^, **SAA**^**^#^**^, sICAM1, sICAM3^*^, sVCAM1, P-selectin, E-selectin, Thrombomodulin	(70)
No significant differences were observed in basal inflammatory state between lean and MetS subjects. Inflammatory resilience was reflected in diminished cytokine and oxylipin responses.	4 week high-fat, high-caloric diet intervention (*n* = 10) in males between 35 and 65 years High fat challenge test dynamics up to 8 h	CRP, SAA, IL-1β, IL-6, **IL-8**^**^#^^a^**^, IL-10, IL-12p70, IL-18, TNF-α, **IFN-γ^#^^b^**, C3, **sICAM1**^#^^b^, sVCAM1, adiponectin^^*^^#^^b^^, leptin^*^^#^^b^, resistin^*^^#^^a^9(S)-HODE, 13(S)-HODE, **11(S)-HETE**^**^#^^a^**^, **12(S)-HETE**^**^#^^a^**^, 9,10-DiHOME, 12,^13^-DiHOME, 19,20-DiHoPE^*^^, *^#^^b^*^, 8,9-DiHETrE, 11,12-DiHETrE, 14,15-DiHETrELeukocytes, **lymphocytes**^**^#^^b^**^, **neutrophils**^**^#^^b^**^, **monocytes**^**^#^^b^**^, eosinophils, basophils	(51)
Fasting levels of immune cell attraction markers were modified with 4-week dark chocolate consumption. No difference between flavonol-enriched and normal dark chocolate.	4-week dark chocolate consumption (*n* = 41) by males with age 45–70 years High fat challenge test dynamics up to 4 h	CRP, SAA, IL-1β, **IL-6**^^#^^, **IL-8**^^#^^, TNF-α^*^^^#^^, sICAM1^*^^^#^^, **sVCAM1**^^#^^, E-selectin, **P-selectin**^**^#^**^, sICAM3^*^^^#^^, Thrombomodulin, vWFLeukocytes^*^^^#^^, **lymphocytes**^**^#^**^, **monocytes**^**^#^**^, **neutrophils**^**^#^**^${\text{CD66b}}_{N}^{*}$, **CD11b**_**N**_^^#^^, **CD62L**_**N**_^^#^^, **CD11c**_**M**_^^#^^, **CD11b**_**M**_^^#^^, **CD62L**$M_{*}^{\#}$, ${\text{CD11c}}_{L}^{\#}$, ${\text{CD11b}}_{L}^{*}$, ${\text{CD62L}}_{L}^{*}$	(87)
Inflammatory resilience was found positively influenced by the anti-inflammatory mix	5 week intervention with anti-inflammatory dietary mix (*n* = 36) of males aged 55–59 years High fat challenge test dynamics up to 6 h	With proteomics profiling, the following markers were significantly changed: **CD40**^**^#^^a^**^, VCAM1^^*^^^,^ *^^#^^*^^a^^**, fibrinogen**^**^#^^a^**^**, EGFR**^**^#^^a^**^**, vWF**^**^#^^a^**^**, ACE**^**^#^^a^**^ **and ICAM1**^**^#^^b^**^, Il-18^^*^^#^^b^^**, TNF RII**^**^#^^b^**^**, adiponectin**^**^#^^b^**^**, factor VII**^**^#^^b^**^**, CCL-22**^**^#^^b^**^, **β2-microglobulin**^**^#^^b^**^	(67, 79)

Markers that were statistically significantly affect by the intervention are shown in bold.

* *marks a difference in fasting levels*,

# *indicates different postprandial challenge test levels*,

a indicates a different challenge response and

b *indicates different offset levels observed due to repeated measurements*.

Altogether, only a few studies have employed a metabolic challenge test to evaluate the effects of nutritional interventions on inflammatory status. They indicate that post-challenge effects may reveal intervention effects on the adaptive and regulatory processes that derail early in the progression toward an unhealthy phenotype. Future applications of metabolic challenges are envisioned to increase insight into dynamics of inflammation to reveal intervention effects on a state of low-grade chronic inflammation.

## Strategies to Improve Inflammatory Dynamics

**Figure 6:**
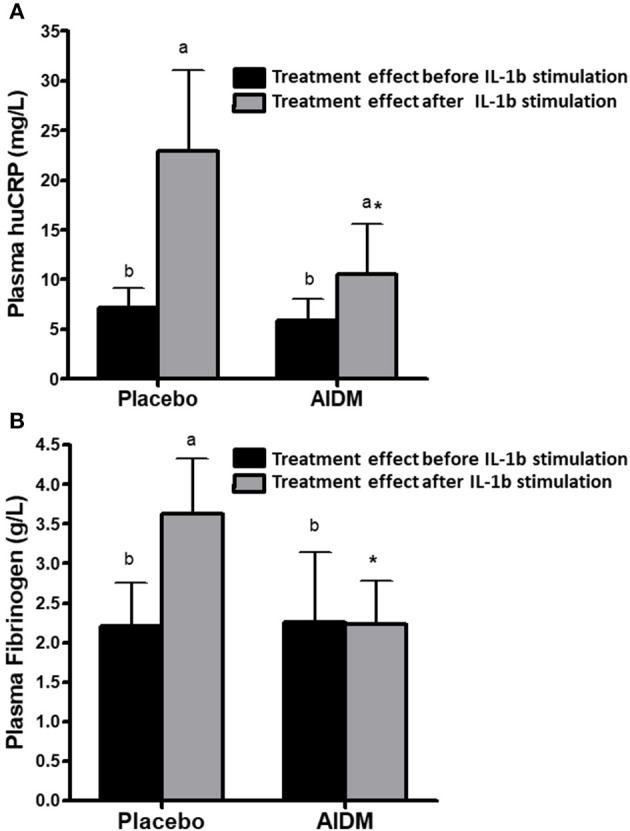
The effect of an “anti-inflammation dietary mixture” (AIDM) vs. placebo quantified at fasting and after Il-1B stimulation on humanCRP **(A)** and fibrinogen **(B)** in transgenic mice. Mice were fed the AIDM and the inflammation markers were quantified after 6 weeks of treatment. The effects of AIDM vs. placebo were only visible after IL1B stimulation and not at fasting, showing the added value of applying a perturbation test to show treatment effects on inflammation. Adapted with permission from Verschuren et al. (88).

As mentioned previously, a metabolic challenge test quantifies low-grade chronic inflammation and nutritional intervention effects more precisely than a classical overnight fasting assay. Ideally, an inflammatory challenge test, e.g., by providing volunteers a low dose of bacterial liposaccharides (LPS) or an attenuated infection such as diarrheagenic *E. coli*, may be even better, but practical and ethical objections limit its use in human studies. However, such an inflammatory challenge test in model animals is feasible, and for example, IL1-β injection as an inflammatory challenge test has been used in mouse studies to study nutritional intervention effects on inflammation. Concomitant to the human intervention study, a 6-weeks high fat intervention study using humanCRP transgenic mice, where the AIDM was responsible for a strong decrease of the Il-1β induced humanCRP and fibrinogen as compared to control (Figure 6) (88). A 16-week exposure of female ApoE3Leiden mice on a high-fat diet with AIDM resulted in an almost complete (96%) reduction of atherosclerotic plaques as compared to the control mice on a high-fat diet (88).

**Figure 7:**
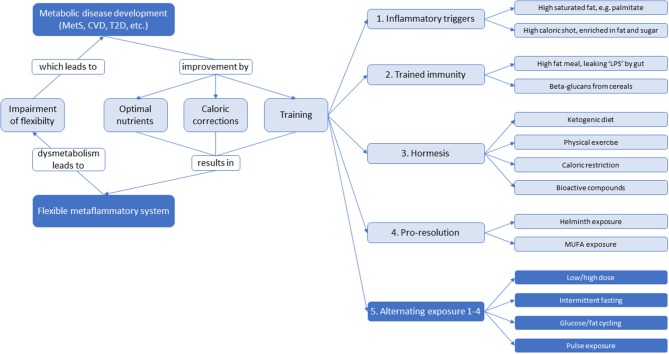
An overview of potential ingredients and strategies for systemic low-grade inflammation therapy to optimize dynamics of inflammation and to maintain a flexible metabolic-inflammatory system. With impairment of flexibility, the system will develop toward metabolic disease development. This can be returned via static nutritional strategies by optimizing nutrients and calories, as well as training strategies through inflammatory triggers, trained immunity, hormesis, pro-resolution, or an alternating combination thereof.

There may also be strategies other than static nutritional interventions focusing on optimizing inflammatory dynamics to reverse or prevent low-grade inflammation. Training of the immune system is a widely accepted biological phenomenon (89–91). The intimate linkage of metabolic pathways and immunity together with the recent progress in our understanding of stress responses as training suggest that metabolism must be trained too to function optimally and flexible. Biological systems which depend on adaptation for their defense, seem to profit from alternating exposure such as low/high dose, intermittent fasting, glucose/fat cycling and pulse exposure (92, 93). For instance, an alternating high-fat dietary regimen reduced systemic insulin resistance, hepatic and renal inflammation, atherosclerosis and improved renal function (92, 94), as well as increased protein content and mitochondrial function in skeletal muscle (95). Although these studies were performed in mice and rats, there is potential for translation to humans given that the processes involved overlap across these mammalian species. An overview of potential ingredients and strategies for training of metaflammation is shown in Figure 7. An effective immune response is characterized by a sequence of counteracting pro- and anti-inflammatory signals that are properly orchestrated to have adequate onset, optimal response, and decay. Each time these mechanisms are challenged by an external trigger (e.g., infections, LPS, extreme physical activity, cellular oxidative stress, inflammatory lipids, …), the immune system is trained to deal with future challenges. This implicates a memory function that is not only observed in the adaptive immune system (which generates memory for specific triggers), but also in the innate immune system, generating a less specific variant of memory referred to as “trained immunity” or “innate immune memory” (89). There are recent indications that the innate immune cells such as monocytes, macrophages, or natural killer cells may be trained. Trained immune cells increase responsiveness upon secondary stimulation by microbial pathogens by expression of so-called pattern recognition receptors (PRRs) that recognize so-called pathogen-associated molecular pattern (PAMPs), such as LPS, flagellin, β-glucan, and lipoteichoic acid (LTA). Initial stimulation with these PAMPs influence long-term production of inflammatory mediators inducing a shift from oxidative phosphorylation toward aerobic glycolysis (the Warburg effect) (89, 96–98). Trained immunity (not to be confused with acute and short-term adaptation mechanisms, such as tachyphylaxis) seems to be a main immunological process leading to non-specific protective effects against infections that persist for weeks to months, however when not properly activated, trained immunity can cause a dysbalanced immune system as in immune paralysis in sepsis, hyperinflammation in tissues, or atherosclerosis (99).

A molecular basis for trained immunity is believed to be an epigenetic-metabolic interplay (89, 98). Epigenetic mechanisms control gene expression via DNA methylation, chromatin remodeling, and microRNA-regulated transcriptional silencing. Cellular metabolites, such as acetyl-CoA, α-ketoglutarate, and succinate act as a cofactor on epigenetic enzymes influencing the functionality of epigenetic mechanisms (89, 96). Indeed, trained immunity seems dependent on the metabolic status of an individual. For example, mevalonate, a precursor of the cholesterol synthesis pathway, was demonstrated to induce trained immunity by epigenetic reprogramming and increased mevalonate levels were associated with higher cytokine levels, while reduction of mevalonate by statins resulted in lower cytokine levels (100).

Although the field of “immunometabolism” has emerged where the relationship between metabolism and trained innate immunity are becoming more mechanistically clear (89), at this stage it is not known for certain whether this training aspect is valid only for external inflammatory triggers (infections, etc.) or for metabolism triggered inflammation and metabolic health/flexibility as well. One could speculate that metabolic triggers known to cause a mild acute inflammatory response improve inflammation dynamics via such mechanism. It is known that saturated fat, especially palmitate (10, 101), high-fat meals (74), calorie-dense meals (66) and meals enriched in fat and glucose (68) all cause an acute inflammatory trigger in healthy individuals. On the other hand, it is also known that diets enriched in mono-unsaturated fat result in reduced postprandial activity of transcription factor NF-kB, protein levels of nuclear p65 and TNF-α (77). It will be interesting to see whether well-scheduled consumption of these type of meals as an inflammatory trigger may improve long-term inflammatory status.

Training aspects have also been described for a process referred to as hormesis. Hormesis is the adaptive reaction of a biological system to external stresses to improve its resilience and tolerance to stronger stresses in the future (90, 102). This involves the observations that low doses of chemicals that plants make as a defense to ward off insects, consumed by humans in low levels via fruit and vegetables, are associated with health benefits. A well-known example is represented by the phytochemical resveratrol. At low doses (2.5 and 5 mg/kg) a reduction of myocardial infarction was seen in rats upon 14 days of consecutive administration, while at high doses (25 and 50 mg/kg), an increase was observed (103). *In vitro* and *ex vivo* studies highlight multiple pathways for which hormesis has been observed in case of resveratrol, including inflammatory pathways (103, 104). Indeed, the secretion of IFN-γ, IL-2, and IL-4 from stimulated human peripheral blood mononuclear cells (PBMCs) demonstrated a clear bi-phasic pattern with increasing doses of resveratrol (104). These apparently strange findings illustrate a highly interesting principle. Relatively low concentrations of anti-inflammatory compounds might actually induce the inflammatory capacity by their pro-inflammatory action, by creating alertness toward these foreign compounds. Thus, instead of a continuous relaxation and “high-dose drug-like” anti-inflammatory condition, these low dose (and often intermittent concentrations) of foreign compounds create a “first line of defense” barrier of inflammation dynamics.

Although this line of research has not yet been thoroughly investigated, numerous anecdotal reports describe the pro- and anti-inflammatory (= hormesis) action of natural compounds (90). For example, livestock in Europe greatly benefitted from inflammatory hormesis by providing multiple mixtures of natural compounds upon the recent ban of antibiotics (105).

These mild stresses from natural compounds induce a memory or training by adapting mammalian physiology to increase their capacity to deal with higher amounts of stress. Other well-known examples of hormesis include calorie-restriction, exercise, and glucose restriction all resulting in longevity in model systems, suggesting that mild induction of oxidative stress within the mitochondria causes an adaptive response that results in increased stress resistance and long-term reduction of oxidative stress (106). Hormesis seems to be induced via epigenetic regulation, which can occur not only during early developmental stages but also through adulthood (107). From a molecular point of view, trained immunity and hormesis may thus be similar stimuli to the immune system. In this hormesis context, the so-called epigenetic diet has been proposed, which introduces bioactive compounds such as genistein, sulforaphane, tea polyphenols, curcumin, resveratrol, vitamins, and minerals that have been demonstrated to act through an epigenetic mechanism resulting in restoration of immune homeostasis thereby supporting healthy aging (108, 109).

A final aspect to consider in the promotion of metaflammation is to stimulate a pro-resolution (Th2 and/or T regulatory) response by changing the environmental hygiene by exposure to pathogens or their products. There is evidence that exposure to pathogens (bacteria, viruses, and helminths) is critical to promote normal immune development, which is reflected in the so-called (revised) hygiene hypothesis (91, 110). Parasitic helminths have been shown to modulate the host immune system and induce responses of Th2 and regulatory immune cells (111, 112). Since pro-inflammatory immune responses play a key role in the development of lifestyle-related diseases such as type 2 diabetes, helminth-induced immunomodulation through controlled infections with helminths or helminth-derived molecules has been investigated and shown to be promising to improve metabolic outcomes in mice (111–115), although at this stage there is very little human data available. Studies indicate that helminths may improve dynamics of inflammation, at least partly, by increasing the number of anti-inflammatory immune cells (Th2 cells, eosinophils, M2 macrophages, Tregs, and ILC2s) in adipose tissue, thereby reducing inflammation and improving glucose tolerance [reviewed by de Ruiter et al. (112)]. Besides changes in adipose tissue, the cellular hepatic composition appeared to be affected by helminth products (113). Furthermore, the helminth-induced increase in ILC2, eosinophils and M2 macrophages may result in browning of white adipose tissue, generating “beige” cells that increase energy expenditure, thereby decreasing adiposity, which may prevent the development of insulin resistance (111, 114, 115). Besides stimulating an anti-inflammatory immune response, helminth infections have been shown in an experimental setting to induce weight loss in mice with diet-induced obesity by impairing glucose absorption (116) or to suppress adipogenesis, thereby preventing the onset of MetS including diabetes (111, 116). In humans, epidemiological cross-sectional studies suggest that chronic helminth infections may prevent development of metabolic diseases and that these effects can be long-lasting [reviewed by de Ruiter et al. (112)]. Longitudinal studies are now required to provide information on the causal relationship between helminths and prevention of metabolic diseases.

## Conclusion

In conclusion, metabolic inflammation should be quantified as a systemic dynamic process. We have presented a state-of-the-art overview of methods to evaluate the inflammatory dynamics of individuals by applying metabolic challenge tests. These dynamics can be optimized by dietary and other interventions, i.e., by exposure to nutrients that improve inflammatory resilience. The first nutritional intervention studies have been published that showed that inflammatory dynamics can be improved (see Table 3), and we envision the next step that focusses on strategies that (re)gain inflammatory flexibility. Literature is providing more and more indications that the inflammatory response may be trained by regular mild inflammatory, as well as metabolic triggers, regulated through epigenetic and metabolic modifications. By combining aspects of metabolic and inflammatory triggers that introduce mild inflammation with knowledge on trained immunity, hormesis and pro-resolution, new strategies to optimize dynamics of inflammation may become available.

## Disclosure

The work was sponsored by Johnson & Johnson Family of Consumer Companies. The sponsor had no input on the design, data, or conclusions of the study.

## Author Contributions

SW, BvO, and WvdB contributed conception of the review. WvdB, JvB, KS, FH, LV, RK, BvO, and SW wrote sections of the manuscript. All authors contributed to manuscript revision, read and approved the submitted version.

### Conflict of Interest Statement

GR, an employee of Janssen Research and Development, LLC, contributed to the revision, read and approved the manuscript, but had no role in the conceptualization and writing of the manuscript. The remaining authors declare that the research was conducted in the absence of any commercial or financial relationships that could be construed as a potential conflict of interest.

## Abbreviations

OGTT: oral glucose tolerance test
MetS: metabolic syndrome
AIDM: anti-inflammatory dietary mixture
LPS: lipopolysaccharide
PBMC: peripheral blood mononuclear cell.
